# Rural Health in Brazil: a still relevant old subject

**DOI:** 10.11606/S1518-8787.2018052000supl1ap

**Published:** 2018-09-13

**Authors:** Carlos E A Coimbra

**Affiliations:** I Fundação Oswaldo Cruz . Escola Nacional de Saúde Pública . Rio de Janeiro , RJ , Brasil

The demographic dynamics of the Brazilian population has undergone important transformations throughout the last century, in particular from the 1940s. These changes have been characterized by sharp declines in fertility and mortality rates, increased life expectancy at birth, and consequently aging of the general population. This scenario was followed, step-by-step, by rapid urbanization, which has turned an eminently rural Brazil into a country where approximately 85% of its population lives in urban areas ^1^ .

The characterization of the rural Brazilian individual as ignorant, ragged, chronically ill, anemic, and lazy was emblematic in the debates about the socioeconomic, health, and political backwardness of the country in the first decades of the twentieth century. This individual was personified as Jeca Tatu, created by the writer Monteiro Lobato ^2^ and made public in the first edition of the short story collection *Urupês* , in 1918. Even though he is a character in the literature, Jeca Tatu became a key element in social and political debates about the project of the nation. Over time, Jeca Tatu was widely interpreted and reinterpreted in the intellectual, medical-scientific, and political circles, committed to social causes, which called for education and sanitation as fundamental strategies to leverage the development of the country.

The year of 2018 marks the centenary of Jeca Tatu’s birth. As expected, a century later, the Brazil of today is very different from that when Lobato conceived one of his best-known creations. However, the allusion to this important character of the Brazilian literature can help us reflect on socio-historical issues that are still present at some level; when compared to the segment of the Brazilian population living in urban areas, there are still important gaps in the knowledge about the social, cultural, and environmental determinants of the health of Brazilians living in rural areas.

On the one hand, it is important to recognize that contemporary Brazil has achieved great successes in the fields of education and rural health. It has practically freed its rural population from a range of infectious and parasitic diseases that are now preventable through vaccines and other primary care strategies. Today, a young student in the health area will hardly be confronted with a case of yaws (which plagued the Brazilian Northeast until the middle of the 1950s) ^3^ , bancroftian filariasis (today rare and occurring only in a few foci), and acute Chagas disease in children ^4^ , as well as severe forms of diarrhea and acute infant malnutrition (kwashiorkor, marasmus), which were common in pediatric wards until a few decades ago ^5^ .

On the other hand, if this trajectory of transformations is relatively well known, what can we say about the Brazilian individuals who live in rural areas today and what challenges do they face in order to keep their health, nutrition, and food security? Regardless of their ethnic or racial identity, little is known about them. Taking into account the fact that large national health diagnoses rarely contemplate rural population strata in their sample plans, any attempts to generalize the health of indigenous peoples, *caiçaras* , *caboclos* , *ribeirinhos* , *caipiras* , *quilombolas* , *pantaneiros* , *boias-frias* , artisanal miners, among many other populations is limited.

As recognized by the Brazilian Ministry of Health itself, in order to achieve effective health actions or develop adequate public policies in rural areas, it is necessary to know the population living there, considering their cultural, social, and environmental specificities ^6–8^ . However, few publications evaluate the general conditions or more than one health indicator of the rural population at the national or regional level ^9^ . Most publications focus on specific aspects of the health of rural workers, commonly associated with occupational health and toxicology ^10^ , or endemic parasitic diseases, such as malaria in the Amazon ^11^ ^,^ ^12^ and schistosomiasis in the Northeast region ^13^ . Population-based studies that consider multiple health outcomes and a specific ethnic context are uncommon, and we highlight that over the last decade research on the health status of indigenous and *quilombola* populations has been expanded, to mention two examples ^14–16^ .

In this sense, this supplement of the *Revista de Saúde Pública* (RSP), based on research coordinated by professors Helen Gonçalves, Elaine Tomasi, Maria Cecília Assunção, and Luciana Tovo-Rodrigues of the Graduate Program in Epidemiology of the *Universidade Federal de Pelotas* , and edited by Helen Gonçalves and Euclydes A. Castilho, arrives in good time. In addition to filling an important gap in the Brazilian scientific literature in the field of rural health, it addresses the topic focusing on case studies in Southern Brazil, one of the most urbanized areas in the country.

The supplement brings together results from a population-based survey on the health of adults aged ≥ 18 years in the rural area of the city of Pelotas, state of Rio Grande do Sul. In eight articles, the set of authors competently addresses the methodological challenges characteristic of research carried out with rural populations in Brazil in general, including dispersion of the population, difficulty in accessing households, underreporting of births and deaths, as well as low and inconsistent coverage of primary health care services, which limits the available information about the main causes of illness and death of the population.

Depressive symptoms, sleep disorders, quality of the diet, general and abdominal obesity, physical inactivity, alcoholism, smoking, low quality of life, and dissatisfaction with health are the main health issues addressed by the contributors of this supplement. According to the authors of one of the articles, “[...] the most relevant aspects that negatively defined the quality of life of the population were being a woman, older, non-white, having a low income, having a lower education level, having always lived in the rural area, being unemployed, and having a disease” ^17^ .

The health scenarios that emerge from the rural area of Pelotas, one of the southernmost cities of the country, may seem particular at first, but this impression is undone after reading the articles. The report of the authors delineates a close picture of that unequal Brazil that we know, in which gender, race/ethnicity, education level, and socioeconomic class play an important role in determining morbidity and mortality, as well as the interpretation of the authors on the perceptions that individuals have about their own well-being and health.

Returning to Jeca Tatu, the transformation he underwent under his creator was as significant as his appearance in 1918. Lobato himself reviewed his pessimism about Jeca Tatu, recreating him from another perspective in the 1920s, signaling that Jeca Tatu “was not born like that,” but “he was like that at the moment.” That is, the socioeconomic, structural, and political conditions were the main determinants of his precarious social and health situation.

I am sure that this supplement of the RSP will gladly fulfill the dual mission of not only informing the particular context of persons living in the rural area of Pelotas but also provoking the Brazilian collective health community into devoting more attention to the rural population of the country as a whole. The understanding of why the health situation of rural populations “is like that” is a fundamental step in the design and implementation of more adequate public policies in a country that remains largely unknown and neglected, even in the most researched and known regions, such as the South.
